# Study on the correlation between homocysteine-related dietary patterns and gestational diabetes mellitus:a reduced-rank regression analysis study

**DOI:** 10.1186/s12884-022-04656-5

**Published:** 2022-04-10

**Authors:** Yu-hong Liu, Ling-peng Lu, Min-hui Yi, Chun-yan Shen, Gu-qin Lu, Jie Jia, Hui Wu

**Affiliations:** 1 Department of obstetrics and gynecology, Seventh People’s Hospital of shanghai University of Traditional Chinese Medicine, Shanghai, 200137 China; 2 Department of Clinical lab, Seventh People’s Hospital of shanghai University of Traditional Chinese Medicine, Shanghai, 200137 China; 3 Department of Nutrition, Seventh People’s Hospital of shanghai University of Traditional Chinese Medicine, Shanghai, 200137 China; 4 Department of Nutrition, School of Medicine Shanghai Jiao Tong University, Shanghai, 200137 China

**Keywords:** Homocysteine, Dietary patterns, Gestational diabetes mellitus, Reduced rank regression, Clinical study

## Abstract

**Background:**

This study aimed to evaluate the association between homocysteine-related dietary patterns and gestational diabetes mellitus.

**Methods:**

A total of 488 pregnant women at 24–28 weeks of gestation between January 2019 and December 2020 were included. Demographic characteristics, dietary intake, and multivitamin supplement intake information were collected using a food frequency questionnaire (FFQ); fasting venous blood samples were collected for serum index detection. Serum homocysteine (Hcy), folic acid, and B12 were selected as response variables, and hyperhomocysteinemia (hHcy)-related dietary patterns were extracted using the reduced rank regression.. The relationship between the score of hHcy-related dietary patterns and GDM was analyzed using a multivariate logistic regression model.

**Results:**

Three hHcy-related dietary patterns were extracted. Only mode 2 had a positive and significant relationship with the risk of developing GDM. After adjusting for confounding factors, the risk of GDM was significantly increased in the highest quartile array compared with the lowest quartile of the pattern (OR = 2.96, 95% Confidence Interval*:* 0.939–9.356, *P* = 0.004). There was no significant correlation between dietary pattern 1 and GDM risk (*P* > 0.05).

**Conclusions:**

Homocysteine-related dietary patterns were positively associated with gestational diabetes mellitus. Adjusting dietary patterns may contribute to the intervention and prevention of GDM.

## Background

Due to the increase in the incidence of obesity and elderly parturients, the incidence of gestational diabetes mellitus (GDM) in mainland China is 14.8%, whereas East China, Central China, and North China have a higher prevalence [1]. The prognosis of GDM mainly depends on early prevention and intervention, among which dietary therapy is an important strategy for its primary prevention and is the basis of all diabetes treatments [2]. Dietary pattern is a comprehensive evaluation of diet as a whole, which can accurately reflect the effect of diet on diseases [3] and is significant in the study of the relationship between nutrition and health [4]. Studies have found that high homocysteine levels (hyperhomocysteinemia, hHcy) are risk factors for GDM [5], and dietary patterns affect serum homocysteine (Hcy) levels; For example, a Mediterranean diet [6] and frugal diet [7] can significantly reduce serum Hcy levels. However, whether dietary patterns affect the incidence of GDM through changes in Hcy levels and its related mechanisms remains unelucidated.

There are three types of dietary pattern-extraction methods, different methods were used according to the purpose of the study [8]. First is the use of dietary indices (DI), including dietary balance index (DBI), dietary quality index (DQI), healthy dietary index (HEI). It is mainly used to evaluate the dietary nutritional status of groups and individuals. For example, Pei-Yan Chen et al. used CHEI’s method to evaluate the dietary status of 1440 patients with primary liver cancer in compliance with the recommendations of the 2016 Chinese Dietary Guidelines [9]. Second is Clustering analysis, which includes principal component analysis (PCA), cluster analysis (CA), and factor analysis (FA) Clustering analysis is a statistical method to classify individuals or variables according to their characteristics. In the case of unclear classification of the overall category, clustering analysis can be used to explore the classification of individuals or variables. For example, Villegas R et al. conducted a follow-up study on 64,191 middle-aged women in Shanghai, clustered their diet into three categories by clustering analysis method, and studied the relationship between these three dietary patterns and type 2 diabetes [10]. Third is the combination of methods from the first and second classes, which includes reduced rank regression (RRR) and partial least-squares regression (PLS). PLS is mainly suitable for linear retrospective modeling of multiple dependent variables and multiple independent variables. It is rarely used alone. It is often used in comparison with other methods to explore the characteristics of dietary patterns of objects and the relationship between them and health outcomes [11]. RRR method can explain the reaction variables to the greatest extent, such as variables related to disease outcomes and nutrients, explain the variations [8], contribute to the analysis of the relationship between dietary patterns and disease, and analyze its possible mechanism. The applications of RRR are expanding in the field of nutrition epidemiology [12]. Therefore, this study aimed to extract hHcy-related dietary patterns using the RRR method and analyze their relationship with the incidence of GDM to explore the possible role of dietary patterns in Hcy levels and GDM.

## Methods

### Subjects

The subjects were pregnant women at 24–28 weeks of gestation. Inclusion criteria were:. singleton pregnancies, Junior high school or above, able to understand the survey content accurately, and willing to accept the questionnaire. Exclusion criteria were: risk factors for diabetes include:family history of diabetes,history of gestational diabetes, history of fetal macrosomia delivery, history of recurrent spontaneous abortion, history of recurrent candidal vaginitis, have abnormal glucose tolerance. This study was approved by the Medical Ethics Committee of the Seventh People’s Hospital Affiliated to Shanghai University of Chinese Medicine (ethics batch number: 2019-7th-HIRB-014). All the study participants provided informed consent.

### Research methods

General demographic characteristics: Sociodemographic data (age, education, and gestational age) and pregnancy history (number of pregnancies/births) were collected by trained investigators. Height and weight were measured using uniform standards and specifications, and body mass index (BMI) before pregnancy was calculated to record weight gain during pregnancy.

Dietary questionnaire survey: Dietary review method and food model were used to collect dietary intake and multivitamin supplement intake of pregnant women from conception to the present,through face-to-face interviews with Food Frequency Questionnaire (FFQ),. At the same time, pregnant women were asked about their intake of nutritional supplements, and their dietary intake of folic acid, vitamin B6 and vitamin B12 was recorded according to the product’s content of nutritional supplements.. According to the food classification principles in the Chinese Food Composition List (sixth edition) [13], food types are classified and sorted into 24 types of food groups. All food intake data were standardized using NutritionStar software (Yingkang Technology Company). The specific method was as follows: the intake dose of each food group was equal to each intake multiplied by the daily intake frequency. Food with an intake dose proportion ≤ 5% (crab/shell, egg tarts/shaomai, coffee/tea, and condiments) were not included in the dietary pattern analysis, because when food intake dose proportion ≤ 5% cannot be included in the analysis of factor loading, so there is no statistical significance in analysis of RRR. In addition, subjects whose intake frequency of the 24 food groups was > 99% with an energy intake of < 800 kcal were excluded,because these subjects had errors in questionnaire answers or survey bias. Finally, 488 cases were included in the analysis.

Diagnostic criteria for gestational diabetes: Pregnant women were screened for gestational diabetes mellitus at 24–28 weeks of gestation (referred to as “glucose screening”). Glucose screening was a 75 g oral glucose tolerance test (OGTT) according to China’s Guidelines for the Diagnosis and Treatment of Gestational Diabetes Mellitus (2014) [14]. GDM is diagnosed if the blood glucose level reaches or exceeds the lower limit as follows: Fasting blood glucose (FBG) 5.1 mmol/L, 1-h postprandial blood glucose (1 h PG) 10.0 mmol/L, and 2-h postprandial blood glucose (2 h PG) 8.5 mmol/L. According to the results from the OGTT, patients were divided into normal groups(*n* = 345) and GDM groups(*n* = 143).

Serum index detection: The blood glucose in the OGTT was measured using the hexokinase method with a Beckman automatic biochemical analyzer (AU5800). Serum Hcy was detected using the enzyme cycle method with a Beckman automatic biochemical analyzer (AU5811),the normal range of Hcy is 0–15.0 umol/L. Serum folic acid (FA) and B12 folic acid were determined by the chemiluminescence method using an Abbott Automatic Immunoanalyzer (I1000S),the normal range of FA is 7.00–46.40 nmol/L,the normal range of B12 is 138.00–652.00 pmol/L. Quality control was performed for all tests prior to testing. When the quality was not controlled, the specific reasons were analyzed and dealt with accordingly.

### Statistical methods

Differences between the GDM and normal groups were compared using the t-test or χ^2^ test. RRR analysis was performed with the option (METHOD = RRR) in the PLS process of SAS software version 9.4 (SAS Institute, North Carolina, USA). Serum Hcy, FA, and B12 values were used as response variables, and as the RRR method could obtain at most the same number of dietary patterns as the number of response variables, three dietary patterns explaining hHcy variation could be obtained in this study. The dietary pattern factor load represented the size and direction of each food group’s contribution to hHcy-related dietary patterns, and the dietary pattern score was obtained by multiplying the dietary pattern factor load by the standardized food intake. The relationship between the scores of the three dietary patterns and the intake of each food group was evaluated using Pearson’s correlation. The subjects were divided into four groups according to the quartile of dietary pattern score, the characteristics of the subjects were analyzed, and a trend analysis was performed. The quartiles of dietary scores were used as independent variables, and logistic regression was used to analyze the relationship between hHcy-related dietary pattern scores and GDM after adjusting for age, educational background, gestational grade, BMI before pregnancy, weight gain during pregnancy, energy intake, and multivitamin intake.

## Results

### General features

A total of 512 pregnant women at 24–28 weeks of gestation who underwent regular obstetric examinations at our hospital between January 2019 and December 2020 were included in the study. Finally, 488 cases were included in the analysis. There was no difference in educational background and weight gain during pregnancy in the GDM group compared to the normal group (*P* > 0.01). However, patients in the GDM group were older, the multiparous women was higher, and the pre-pregnancy BMI and energy intake levels were higher (*P* < 0.01). The intake of folate in the GDM group was lower than that in the normal group, but there was no difference in the intake of B12 and B6 between the two groups (*P* > 0.01). Serum Hcy levels were higher in the GDM group, but FA and B12 levels were lower in the GDM group than in the normal group (*P* < 0.01) (Table 1).

**Table 1:** Comparison of basic characteristics between the GDM and normal groups

Essential characteristics	OGTT		*t*-value	*P*-value
	normal group (*n* = 345)	GDM group (*n* = 143)		
Age	28.513 ± 4.447	30.636 ± 4.645	4.739	< 0.001
Educational background			−0.576	0.565
Senior high school and below	188 (54.50)	82 (57.34)		
College degree or above	157 (45.50)	61 (42.66)		
Gravidity			−2.475	0.013
Primipara	180 (52.17)	57 (39.86)		
Multipara	165 (47.83)	86 (60.14)		
Pregnancy BMI (kg/m^2^)	22.097 ± 3.629	23.992 ± 4.527	4.450	< 0.001
Weight gain during pregnancy (kg)	8.774 ± 4.144	8.794 ± 4.992	0.045	0.964
Energy intake (kcal/d)	1464.085 ± 411.720	1642.900 ± 636.197	3.103	0.002
Multivitamin supplement intake				
Dietary FA (ug)	538.936 ± 572.832	431.846 ± 445.723	1.998	0.028
Dietary B12 (ug)	2.631 ± 6.828	2.472 ± 9.833	0.204	0.838
Dietary B6 (mg)	1.974 ± 2.288	1.772 ± 2.502	0.862	0.389
Serological indicator				
serum FA (nmol/L)	21.967 ± 10.523	18.189 ± 14.161	2.877	0.004
serum B12 (pmol/L)	219.294 ± 110.621	188.632 ± 99.506	2.997	0.003
serum Hcy (umol/L)	5.770 ± 1.668	7.235 ± 5.007	3.423	< 0.001

Note: Categorical variables include educational background and pregnancies, expressed as the number of people (constituent ratio). Continuous variables included age, pre-pregnancy BMI, weight gain during pregnancy, energy intake, intake of multivitamin supplements (FA, B12, and B6), and serological indicators (FA, B12, and Hcy), expressed as mean ± standard deviation, in which energy intake did not include the energy provided by cooking oil intake

### Characteristics of RRR dietary pattern

Three dietary patterns were identified in the present study (Table 2). For mode 1, the correlation index was > 0.20 mainly for poultry meat and livestock meat; and < − 0.20 for green leafy vegetables, dark vegetables, soybeans, and shrimp, which explained the 29.14% variation in food and 24.26% variation in response variables. For mode 2, the correlation index was > 0.20 mainly for cooked wheaten food, livestock meat, and eggs; <− 0.20 mainly for coarse cereals, green leafy vegetables, dried fungi and algae, milk Group and nuts, which explained the 65.23% variation in food and 56.38% variation in response variables. The correlation index of mode 3 factors was > 0.20 mainly for livestock meat; <− 0.20 for soybeans, which explained the 5.63% variation in food and 19.35% variation in response variables. As the explanation variation of mode 3 was relatively small, it was excluded.

**Table 2:** The load of each food component in hHcy-related dietary pattern and its correlation with dietary pattern score

	Pattern 1		Pattern 2		Pattern 3	
Food group	factor loading	correlation index	factor loading	correlation index	factor loading	correlation index
Cooked wheaten food	−0.077	− 0.142^c^	0.231^a^	0.564^c^	0.015	0.057
Coarse cereals	−0.023	− 0.048	− 0.316^a^	−0.222^c^	− 0.040^a^	−0.048
Green leafy vegetables	−0.222^a^	− 0.160^c^	− 0.233^a^	−0.559 ^c^	0.040	0.014
Dark vegetables	−0.261^a^	− 0.113^b^	0.046	− 0.426 ^c^	0.010	0.082
Dried fungi and algae	0.143	0.131 ^c^	−0.232^a^	−0.480 ^c^	0.022	0.010
High energy fruits	0.130	0.600 ^c^	−0.056	−0.505 ^c^	− 0.021	0.059
Poultry meat	0.311^a^	−0.168^c^	0.087	0.470 ^c^	0.038	0.029
Livestock meat	0.251^a^	−0.030	0.269^a^	0.461 ^c^	0.237	0.239 ^c^
Fish	0.160	0.244 ^c^	0.041	− 0.386 ^c^	− 0.080	0.188 ^c^
Shrimp	−0.242	0.070	0.062	−0.273 ^c^	0.022	0.005
Eggs	0.178	−0.048	0.291^a^	0.515 ^c^	0.066	0.065
Milk Group	− 0.074	− 0.256 ^c^	− 0.247^a^	0.107 ^b^	0.020	0.064
Soybeans	−0.212^a^	0.006	−0.112	− 0.432 ^c^	− 0.281 ^a^	−0.614 ^c^
Nuts	0.178	0.236 ^c^	−0.577^a^	−0.436 ^c^	0.059	0.088 ^c^
Explain the proportion of variation	Pattern 1	Pattern 2	Pattern 3	summation		
Explain each food group	4.250	9.512	0.821	14.583		
Explanatory response variable	0.361	0.839	0.288	1.488		

Note: ^a^Only food groups with absolute factor load > 0.20 are shown. ^b^*P* < 0.05, ^c^*P* < 0.01

### Characteristic analysis of dietary pattern quartile

Compared with the lowest quartile array of pattern 1, the subjects in the highest quartile array of pattern 1 had higher energy intake, higher serum Hcy, higher serum FA and B12, and both showed a linear trend. Compared with the lowest quartile array of pattern 2, the subjects in the highest quartile array were older, had higher pre-pregnancy BMI, higher serum Hcy, and lower serum FA and B12, with a linear trend, but there was no difference in energy intake and weight gain during pregnancy (Table 3).

**Table 3:** Characteristics of subjects under different quartile scores of hHcy-related dietary patterns

	Pattern 1			Pattern 2		
	Q1	Q4	*P* values	Q1	Q4	*P* values
Age	29.254 ± 4.203	28.869 ± 4.722	0.502	28.746 ± 4.512	30.508 ± 4.679	0.0046
Educational background			0.051			0.054
Senior high school and below	77(63.64)	61(50.41)		54(44.63)	69(57.02)	
College degree or above	44(36.36)	60(49.59)		67(55.37)	52(42.98)	
Gravidity			0.062			0.072
Primipara	57(47.11)	72(59.50)		70(57.85)	55(45.45)	
Multipara	64(52.89)	49(40.50)		51(42.15)	66(54.55)	
Pregnancy BMI (kg/m^2^)	22.978 ± 4.088	22.078 ± 3.393	0.063	21.836 ± 3.488	23.804 ± 4.355	< 0.001
Weight gain during pregnancy	8.863 ± 4.782	9.502 ± 4.337	0.276	7.947 ± 4.082	8.930 ± 5.365	0.1084
Energy intake (kcal/d)	1482.1 ± 479.3	1523.3 ± 468.7	0.002	1510.9 ± 508.5	1530.0 ± 550.2	0.7786
Multivitamin supplement intake						
dietary FA (ug)	516.10 ± 637.70	477.20 ± 411.10	0.574	518.90 ± 537.60	498.90 ± 557.90	0.778
dietary B12 (ug)	2.086 ± 3.370	2.952 ± 10.550	0.394	2.153 ± 2.917	2.210 ± 2.980	0.882
dietary B6 (mg)	1.770 ± 2.070	2.010 ± 2.636	0.433	1.830 ± 1.811	1.760 ± 1.866	0.768
Serological indicator						
serum FA (nmol/L)	183.6 ± 110.900	219.5 ± 82.669	0.0008	238.7 ± 96.952	115.7 ± 62.542	< 0.001
serum B12 (pmol/L)	17.752 ± 11.976	22.443 ± 9.465	0.0046	23.883 ± 10.075	9.266 ± 6.041	< 0.001
serum Hcy (umol/L)	7.344 ± 3.141	5.403 ± 1.805	< 0.001	5.286 ± 1.337	9.577 ± 2.833	< 0.001

Note: Categorical variables include educational background and pregnancies, expressed as the number of people (constituent ratio). Continuous variables included age, pre-pregnancy BMI, weight gain during pregnancy, energy intake, intake of multivitamin supplements (FA, B12, and B6), and serological indicators (FA, B12, and Hcy), which were expressed as mean ± standard deviation

### Correlation analysis between dietary pattern and GDM

Logistic regression analysis showed that after adjusting for multiple confounding factors, the score of mode 2 was significantly positively correlated with the incidence of GDM (*P* < 0.01), and the risk of GDM significantly increased relative to the lowest quartile array and the fourth quartile array (OR = 2.963,95% CI: 0.939–9.356). However, there was no significant relationship between the score of mode 1 and the incidence of GDM (*P* > 0.05). The risk of developing GDM in the lowest and highest quartiles of the scores was OR = 0.480 (95% CI: 0.137–1.684) (Table 4).

**Table 4:** Logistic regression results of the relationship between hHcy-related dietary pattern score and the prevalence of GDM

Dietary Pattern Score quartiles	Pattern 1		Pattern 2	
	Model 1	Model 2	Model 1	Model 2
Q1	1.000	1.000	1.000	1.000
Q2	0.890(0.461–1.718)	0.809(0.409–1.600)	1.894(0.916–3.917)	1.937(0.911–4.117)
Q3	0.578(0.259–1.290)	0.529(0.230–1.213)	1.983(0.808–4.867)	1.983(0.808–4.867)
Q4	0.518(0.152–1.771)	0.480(0.137–1.684)	2.077(0.812–5.315)^a^	2.963(0.939–9.356)^a^
*P* values	0.350	0.450	0.005	0.004

Note: Model 1: Age, educational background, number of pregnancies, pre-pregnancy BMI, and pregnancy weight gain were adjusted. Model 2: Energy intake and multivitamin supplement intake (folic acid, B12, and B6) were further adjusted. ^a^*P* < 0.01

## Discussion

In this study, serum Hcy,FA and B12 were selected as response variables, and three hHcy-related dietary patterns were extracted using the RRR method. These three dietary patterns could explain the variation in serum Hcy,FA, and B12 to the greatest extent from the perspective of food. Similar proportions of variation in response variables have been explained in other clinical studies using the RRR method [15, 16]. The variation explained by mode 3 was significantly smaller than that of modes 1 and 2; thus, it was excluded. It was found that the scores of modes 1 and 2 were positively correlated with the Hcy level, indicating that these two dietary patterns were closely correlated with the Hcy level; this finding is consistent with that of previous studies. Previous studies have found that Hcy levels are significantly correlated with the risk of GDM, which can significantly increase the risk by 20% [5]. Hcy is a sulfur-containing amino acid, an important intermediate in the methionine metabolism process, and any defects that lead to key enzymes or cofactors can result in methionine cycle problems that affect serum Hcy levels and a unit of carbon metabolism-related vitamins, such as vitamin B6, vitamin B12, folic acid, and betaine, which are important coenzymes in the metabolism process. In recent years, an increasing number of studies have suggested that Hcy is closely related to insulin resistance [17], and hHcy should be included in metabolic syndrome [18]. The mechanism is thought to be a result of Hcy being a vascular damaging amino acid that can induce vascular damage and oxidative stress in pancreatic beta cells, leading to disorders of glucose and lipid metabolism [19].

Mode 2 was characterized by a dietary pattern with a higher intake ofcooked wheaten food (including steamed bun, noodles, dumplings), livestock meat and eggs, and less intake of coarse cereals, green leafy vegetables, dried fungi and algae, milk and nuts. Among the two hHcy-related dietary patterns obtained, only mode 2 showed a positive correlation with the prevalence of GDM. This is consistent with previous studies that state that the dietary pattern with high intake of fruits and vegetables, Coarse cereals, and milk is rich in one-carbon unit metabolic-related vitamins, such as vitamin B6, vitamin B12, folic acid, and betaine, which can reduce the blood Hcy level [7]. However, insufficient intake can increase Hcy levels [20]. There are two components of folic acid intake during pregnancy: from multivitamins and from food (animal liver, poultry, eggs, beans, and leafy greens). In China, continuous supplementation of folic acid in the first 3 months of pregnancy and during pregnancy to prevent fetal neural tube defects is a major public health project [21] to ensure successful birth and good childcare [22]. This study found that only 23.30% of the patients started taking folic acid 3 months before pregnancy, 76.70% of the patients started taking folic acid after being pregnant, and 77.13% continued to take folic acid or multivitamins in the second trimester. The results showed that the awareness of taking folic acid supplements during the perinatal period in the Shanghai area is relatively low, and nutritional education for women of childbearing age needs to be strengthened.

Pattern 1 was characterized by a higher intake of poultry and livestock meat and a lower intake of green leafy vegetables, dark vegetables, soybeans, and shrimp. Poultry meat, livestock meat, and other protein foods are rich in methionine, and a high intake of poultry meat or lack of a carbon-unit metabolism-related vitamin will lead to an increase in serum Hcy concentration, which is consistent with previous studies [20]. A prospective clinical study of 681 patients found a significant correlation between meat dietary patterns and the prevalence of GDM [23]. However, this study did not find a significant correlation between dietary pattern score and the prevalence of GDM, this may be because poultry meat offset the risk of GDM caused by elevated serum Hcy levels in other ways. This may be due to the abundance of choline in poultry meat and livestock meat [24], which is another metabolic pathway of Hcy. It can be determined from the one-carbon unit metabolic pathway table that Hcy produces methionine via two methylation pathways: the folate-dependent pathway and the choline/betaine-dependent pathway [25]. The folate-dependent pathway is well known for supplying methyl, while the choline/betaine-dependent pathway has received little attention. Just as a deficiency of folic acid impedes Hcy methylation, individuals who lack choline also have a reduced ability to methylate Hcy, resulting in hHcy [26]. Choline has been suggested as a candidate nutrient intervention for deficient folate intake or metabolic abnormalities [27, 28].

The advantage of this study is that two types of hHcy-related dietary patterns were extracted by the RRR method, which explained the variation in hHcy to the greatest extent, rather than the variation in food. Therefore, if dietary guidance of the population is needed, dietary patterns should be extracted using principal component analysis and other methods. In addition, the relationship between hHcy-related dietary patterns and the incidence of GDM was analyzed, which is of great significance in exploring the relationship between dietary patterns and GDM through Hcy levels. The limitation of this study is that the subjects were from an obstetric clinic of only one hospital and the sample size was small. In addition, dietary surveys may have a recall bias. As a cross-sectional study, the causal relationship between dietary patterns and GDM could not be determined. In conclusion, hHcy-related dietary pattern scores was significantly positively correlated with the risk of GDM, suggesting that dietary pattern can be adjusted to increase the intake of food rich in one-carbon unit metabolism-related vitamins,thereby affecting the level of Hcy may contribute to the intervention and prevention of GDM.

## Data Availability

All data generated or analyzed during this study are included in this published article. The datasets generated during and analyzed during the current study are not publicly available due to there are various data types in this study, including dietary questionnaires, dietary intake data calculated by nutrition software based on dietary questionnaires, and data analysis of rank-decreasing regression, so it is not suitable for public sharing. But the datasets used and/or analyzed during the current study are available from the corresponding author on reasonable request.

## Abbreviations

GDM: Gestational diabetes mellitus
hHcy: Hyperhomocysteinemia
Hcy: Homocysteine
DI: Dietary indices
DBI: Dietary balance index
DQI: Dietary quality index
HEI: Healthy dietary index
PCA: Principal component analysis
CA: Cluster analysis
FA: Factor analysis
RRR: Reduced rank regression
PLS: Partial least-squares regression
BMI: Body mass index
FFQ: Food frequency questionnaire
OGTT: Oral glucose tolerance test
FBG: Fasting blood glucose
1 h PG: 1-h postprandial blood glucose
2 h PG: 2-h postprandial blood glucose
